# Altered fibroblast proteoglycan production in COPD

**DOI:** 10.1186/1465-9921-11-55

**Published:** 2010-05-11

**Authors:** Oskar Hallgren, Kristian Nihlberg, Magnus Dahlbäck, Leif Bjermer, Leif T Eriksson, Jonas S Erjefält, Claes-Göran Löfdahl, Gunilla Westergren-Thorsson

**Affiliations:** 1 Department of Experimental Medical Science, BMC D12 Lund, Lund University, Sweden; 2 Department of Respiratory Medicine and Allergology, Lund University Hospital, Lund, Sweden; 3 AstraZeneca R&D, Lund, Sweden

## Abstract

**Background:**

Airway remodeling in COPD includes reorganization of the extracellular matrix. Proteoglycans play a crucial role in this process as regulators of the integrity of the extracellular matrix. Altered proteoglycan immunostaining has been demonstrated in COPD lungs and this has been suggested to contribute to the pathogenesis. The major cell type responsible for production and maintenance of ECM constituents, such as proteoglycans, are fibroblasts. Interestingly, it has been proposed that central airways and alveolar lung parenchyma contain distinct fibroblast populations. This study explores the hypothesis that altered depositions of proteoglycans in COPD lungs, and in particular versican and perlecan, is a result of dysregulated fibroblast proteoglycan production.

**Methods:**

Proliferation, proteoglycan production and the response to TGF-β_1 _were examined *in vitro *in centrally and distally derived fibroblasts isolated from COPD patients (GOLD stage IV) and from control subjects.

**Results:**

Phenotypically different fibroblast populations were identified in central airways and in the lung parenchyma. Versican production was higher in distal fibroblasts from COPD patients than from control subjects (p < 0.01). In addition, perlecan production was lower in centrally derived fibroblasts from COPD patients than from control subjects (p < 0.01). TGF-β_1 _triggered similar increases in proteoglycan production in distally derived fibroblasts from COPD patients and control subjects. In contrast, centrally derived fibroblasts from COPD patients were less responsive to TGF-β_1 _than those from control subjects.

**Conclusions:**

The results show that fibroblasts from COPD patients have alterations in proteoglycan production that may contribute to disease development. Distally derived fibroblasts from COPD patients have enhanced production of versican that may have a negative influence on the elastic recoil. In addition, a lower perlecan production in centrally derived fibroblasts from COPD patients may indicate alterations in bronchial basement membrane integrity in severe COPD.

## Background

Chronic obstructive pulmonary disease (COPD) is a progressive disease characterized by a reduction in respiratory airflow that is not possible to normalize [1]. The reduced airflow is caused by tissue remodeling, including reorganization of the extracellular matrix (ECM). In bronchi, epithelial dysregulation results in impaired mucocilliary clearance, over-production of mucus, and squamous cell metaplasia. In parallel with this, subepithelial fibrosis is often observed in bronchi and bronchioles. Degradation of alveolar walls (emphysema) is a hallmark of COPD, which limits the area of air-blood exchange and the elastic recoil [2]. Other structural changes in COPD, such as thickening of the airway wall and reticular basement membrane, have been implicated as factors that contribute to reduction in airflow [3,4].

Interestingly, in terms of the turnover of ECM, opposing pathological processes occur in the COPD lung as the ECM is degraded in alveoli and there is excessive deposition of ECM (fibrosis) in bronchi and in bronchioles [5]. The major cell type responsible for production and maintenance of the ECM are fibroblasts. Recently, it was suggested that central airways and alveolar lung parenchyma contain distinct fibroblast populations [6,7]. Fibroblasts from these anatomical sites were found to have different morphology, proliferation, and ECM production. This distinction is important to consider in COPD, as the ECM turnover is different in bronchi and alveoli. A key family of molecules for ECM integrity is the proteoglycans. The production of proteoglycans and other ECM molecules have been reported to be modulated by the profibrotic signal molecule TGF-β [8,9]. Proteoglycans have been shown to be differentially expressed in COPD lungs [10,11]. For example, enhanced alveolar immunostaining of the large proteoglycan versican has been reported in COPD patients [10]. As versican may inhibit the assembly of elastic fibers, it may have a negative effect on the elastic recoil and thereby possibly contribute to the pathogenic development of COPD. Moreover, perlecan, a heparan sulphate proteoglycan, is crucial for basement membrane integrity and reduced perlecan immunostaining has been demonstrated in the lungs of COPD patients [11].

In this study, we hypothesized that altered levels of proteoglycans in COPD lungs may be dependent on dysregulated proteoglycan production in fibroblasts and hence that there are alterations in fibroblast phenotypes in COPD patients compared to control subjects. In particular, we wanted to determine whether enhanced alveolar versican deposition is due to higher versican production by distal fibroblasts. We also wanted to examine whether perlecan production was altered in centrally derived fibroblasts. Thus, we isolated centrally and distally derived fibroblasts from lung explants from COPD patients and from biopsies from healthy control subjects in order to assess proteoglycan production, proliferative potential, and responsiveness to TGF-β_1 _*in vitro*.

## Methods

### Patients

Patients (n = 8) suffering from very severe COPD (GOLD stage IV) who were undergoing lung transplantation at Lund University Hospital were included in the study. The patients had quitted smoking at least 6 months before the lung transplantation. Non-smokers (n = 12) with no clinical history of asthma, (reversibility < 12% after administration of the β_2_-agonist salbutamol (400 μg) and did not respond to methacholine test doses (< 2,000 μg)), or other lung diseases were included as control subjects. This study was approved by the Swedish Research Ethical Committee in Lund (FEK 91/2006 and FEK 213/2005).

### Isolation of cells

Fibroblasts were isolated from explants from COPD patients and from bronchial and transbronchial biopsies from control subjects as previously described [12]. Briefly, biopsies from control subjects were immediately after sampling transferred to cell culture medium (DMEM supplemented with 10% FBS, Gentamicin, PEST, and Ampotericin B (all from Gibco BRL, Paisley, UK)). Specimens from the lung explants were dissected directly after removal from the COPD patients and were immediately transferred to cell culture medium. Bronchial tissue was collected from the luminal side from the same localisation as where bronchial biopsies were taken and were chopped into small pieces. Alveolar parenchymal specimens from explants were collected 2-3 cm from the pleura in the lower lobes, i.e. from the same location as where transbronchial biopsies were taken. Vessels and small airways were removed from the peripheral lung tissues and the remaining tissues were chopped into small pieces. After rinsing, bronchial and parenchymal pieces from explants and biopsies were allowed to adhere to the plastic of cell culture flasks for 4 h and were then kept in cell culture medium in 37°C cell incubators until outgrowth of fibroblasts were observed. Bronchial and parenchymal fibroblasts were then referred to as central and distal fibroblasts, respectively. Experiments were performed at passages 3-6. The cell cultures were continuously stained to verify the mesenchymal identity and to estimate the purity. In the few cases when the cellular staining was less clear then the cell morphology was verified to be representative for the culture as a whole.

### Proliferation assay

Proliferation was determined as previously described [13]. Briefly, cells were plated and fixed after 6, 24, 48, h. Cells were stained with Crystal Violet and cell numbers were quantified indirectly by absorbance at 595 nm on a spectrophotometer plate reader (EL_X_800; Biotek Instruments, Winooski, VT,). Proliferation was defined as absorbance at 48 h divided by the absorbance after 6 h. With this method the amount of adsorbed dye has been shown to be proportional to the cell number recorded on a Coulter counter [14].

### Immunohistochemistry

#### Staining of fibroblasts

Fibroblasts (7000/well) grown overnight were fixed in 4% paraformaldehyde for 15 minutes. Thereafter unspecific binding sites were blocked by 2% BSA-TBS containing 5% goat serum (Vector laboratories, Burlingame, CA) and 0,1% Triton X for 30 minutes. Cells were incubated with primary antibodies: monoclonal mouse antibody against Prolyl 4-Hydroxylase (Acris antibodies, Hiddenhausen, Germany), monoclonal mouse IgM antibody against Vimentin (Santa Cruz Biotechnology, Santa Cruz, CA), monoclonal mouse IgG2a antibody against α-SMA (Dako, Glostrup, Denmark), monoclonal IgG and antibody against SM22-alpha (Abcam, Cambridge, UK), and with secondary antibodies: Alexafluor 488-conjugated goat anti-mouse antibody and Alexafluor 555-conjugated goat anti-mouse antibody (both from Molecular Probes Invitrogen, Eugene, OR). The DNA-binding molecule DAPI (Molecular Probes Invitrogen, Eugene, OR) was used to stain cell nuclei before final mounting (Dako fluorescence mounting medium, Dako, Glostrup, Denmark). Cells were photographed using a TE2000-E fluorescence microscope (Nikon, Tokyo, Japan) equipped with a DXM1200C camera (Nikon, Tokyo, Japan).

#### Tissue staining of proteoglycans

In short, tissue, from same locations as where pieces for cell isolations were taken, was fixed in 4% paraformaldehyde and embedded in paraffin. 5 μm sections were deparaffinized, rehydrated and then pre-treated overnight in buffer containing chondrotinase ABC (Seikagaku, Tokyo, Japan), in 37°C to make epitopes accessible for antibodies. Endogenous peroxidase activity was blocked in 3% hydrogen peroxidase (Merck, Damstadt, Germany) followed by a 30 minutes block with 2% BSA-TBS containing 5% serum raised in the same species as the secondary antibodies used. Furthermore, endogenous avidin and biotin binding sites were blocked (Vector avidin/biotin blocking kit, Vector laboratories, Burlingame, CA) according to the manufacturer's protocol. Sections were incubated with primary antibodies: rabbit polyclonal antibody against versican (Santa Cruz Biotechnology, Santa Cruz, CA), mouse polyclonal antibody against perlecan (Zymed laboratories, San Francisco, CA), goat polyclonal antibody against biglycan (Santa Cruz Biotechnology, Santa Cruz, CA), mouse monoclonal antibody against decorin (Abcam, Cambridge, UK). This was followed by incubation with secondary antibodies: biotin-conjugated goat anti-rabbit (Vector laboratories, Burlingame, CA), biotin-conjugated horse anti-mouse (Vector laboratories, Burlingame, CA), and biotin-conjugated donkey anti-goat (Jackson ImmunoResarch, West Grove, PA). Sections were incubated with avidin and biotin (Vector laboratories, Burlingame, CA) according to the manufacturer's instructions and were developed with DAB (Vector laboratories, Burlingame, CA) to visualize bound antibodies and then counterstained with Mayer's hematoxylin. Sections were photographed using a TE2000-E fluorescence microscope (Nikon, Tokyo, Japan) equipped with a DXM1200C camera (Nikon, Tokyo, Japan).

### Quantification of proteoglycans

Proteoglycan production in fibroblasts was determined as previously described [15]. Briefly, cells were incubated in sulfate-poor Dulbecco's MEM (Gibco BRL, Paisley, UK) supplemented with 0.4% FBS w/wo 10 ng/ml TGF-β1 (R&D Systems, Minneapolis, MN). COPD fibroblasts had a very contractile phenotype and experiments were therefore performed on cell culture plastics coated with 1% collagen-1 (PureCOL; Inamed Biomaterials, Fremont, CA). This modification did not significantly alter proteoglycan production (data not shown). Proteoglycans were quantified by [^35^S]-sulfate incorporation into glycosoaminoglycan side-chains measured on a scintillation counter (Wallac; Perkin Ellmer, Boston, MA). Individual proteoglycans were separated by SDS-PAGE and quantified using densitometry. The contribution of individual proteoglycans to the total proteoglycan content was calculated as the value for each proteoglycan divided by the sum of all the measured proteoglycans (versican, perlecan, biglycan and decorin) by densitometry.

### Statistics

Data are expressed as mean ± SEM. Statistical differences between groups were determined by multiple comparisons using Kruskal-Wallis test. The Mann-Whitney test was used to compare statistical differences between groups. The Wilcoxon signed rank test was used to determine whether TGF-β_1_-stimulated proteoglycan production was different from basal levels. Differences were considered significant at p < 0.05. All analyses were performed using GraphPad Prism software version 4.00 (GraphPad Software, San Diego, CA).

## Results

### Study subjects

Characteristics of COPD patients and control subjects are shown in Table 1. Predicted FEV_1 _was 19.7% (14-24%); (mean and range) in COPD patients. The corresponding numbers were 106.7% (95-116%) for control subjects. Mean age was 62 years (53-66) in the COPD group. For control subjects the mean age was 30 years (24-41). All the COPD patients were ex-smokers whereas none of the controls had a history of smoking. Distal fibroblast cultures were obtained from all patients and control subjects, while central fibroblasts were obtained from 6 of 8 COPD patients and 7 of 12 control subjects.

**Table 1:** COPD patients and control subjects in the study

Characteristics	Controls		COPD	
No.	12		8	
Age (range)	30	(24--41)	62	(53--66)
Pack years (range)	0		41	(25--60)
Gender, M/F in %	42/58		37/63	
				
**Lung function**				

FEV_1_	4.0	(3.0--5.4)	0.57	(0.4--0.9)
FEV_1 _% predicted	106.7	(95--116)	19.7	(14--24)
FVC	4.8	(3.5--6.4)	2.0	(1.3--2.8)
FEV_1 _% predicted/FVC	22	(17--28)	31	(20--39)
DLCO	m		1.4	(1.4--1.5)^†^
DLCO % predicted	m		24	(14--42)^§^

^† ^Only values from 4 patients. ^§ ^Only values from 5 patients. m denotes that data is missing

Data is presented as mean (range)

### Qualitative evaluation of proteoglycan localization

**Figure 1:**
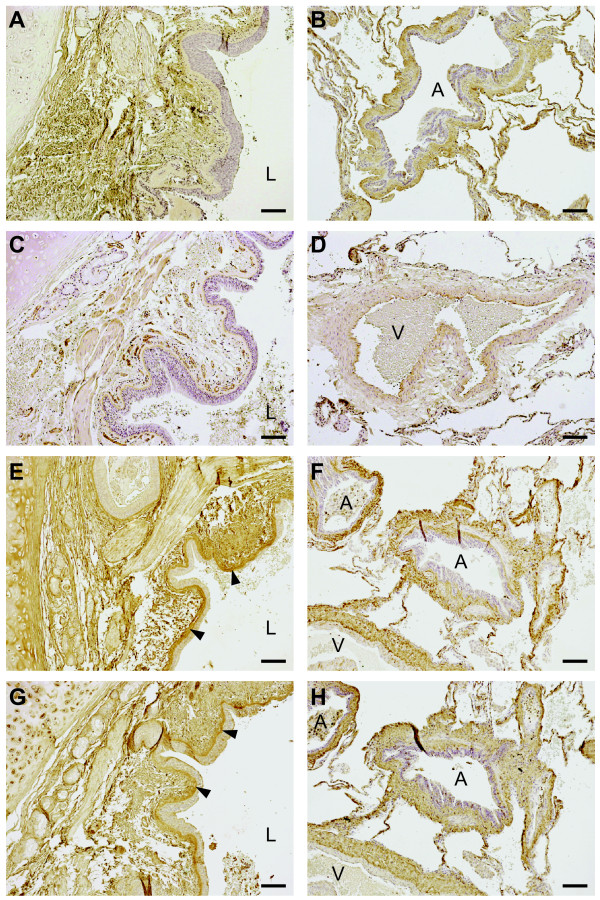
**Proteoglycan staining in lung sections from COPD patients**. Representative micrographs of lung sections from one COPD patient. Antibodies were visualized by DAB staining (shown in brown) and sections were counterstained with Mayer's hematoxylin, which stains cell nuclei blue. **A**, **C**, **E**, and **G **(left panel) are representative micrographs from the central airways (bronchi) and **B**, **D**, **F**, and **H **(right panel) show the small airways and parenchyma. **A **and **B **show versican, **C **and **D **perlecan, **E **and **F **biglycan, and **G **and **H **decorin. L denotes lumen of the bronchi, and A and V denote airway and vessels, respectively. Black arrowheads show staining in the lamina reticularis. Scale bars represent 100 μm.

The localization of versican, perlecan, biglycan, and decorin staining from one representative COPD subject is presented in Figure 1. Immunoreactivity for perlecan was, as expected, identified in the basement membrane of bronchi, bronchioles and blood vessels. Unexpectedly, the bronchial and bronchiolar reticular basement membranes showed immunoreactivity for biglycan and decorin. The basement membrane of pulmonary vessels were positive for decorin but not for biglycan. The lamina propria tissue between basement membranes and smooth muscle layers in bronchi and bronchioles was positive for versican, biglycan, and decorin. Immunoreactivity for these three proteoglycans was also observed in the tunica media of pulmonary arteries, as well as in the adventia of both bronchioles and arterioles. Staining was also present in alveolar walls. Finally, smooth muscle cell layers circumscribing bronchi and bronchioles were slightly positive for perlecan, decorin and biglycan.

### Characterization of fibroblasts

**Figure 2:**
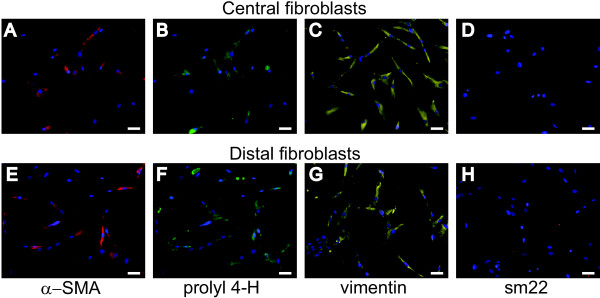
**Immunostaining of COPD fibroblasts**. Isolated fibroblasts were immunostained to verify their mesenchymal origin. Cell nuclei are visualized by DAPI staining, shown in blue. **A--D **(upper panel): representative micrographs of central fibroblasts. **E--H **(lower panel) show distal fibroblasts. Antibodies to α-SMA were used in **A **and **E**, antibodies to prolyl 4-hydroxylase in **B **and **F**, antibodies to vimentin in **C **and **G**, and antibodies to sm22 in **D **and **H**. Scale bars represent 50 μm

Isolated fibroblasts from COPD patients and control subjects were characterized using antibodies to mesenchymal markers (Figure 2). Both central and distal fibroblasts were positive for α-smooth muscle actin (α-SMA), as shown in Figure 2A and 2E. Furthermore, central and distal fibroblasts showed immunoreactivity for the fibroblast markers prolyl 4-hydroxylase and vimentin (Figure 2B, C, F and 2G). The contractile protein Sm22 has been found to be expressed by smooth muscle cells and myofibroblasts but not by fibroblasts [16]. The isolated fibroblasts were negative for sm22.

### Fibroblast proliferation

**Figure 3:**
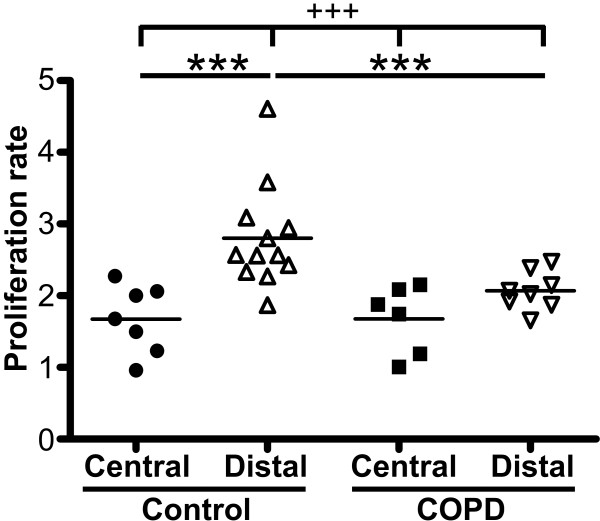
**Proliferation potential of isolated fibroblasts**. Proliferation potential was determined by crystal violet assay, as described in Methods. The data presented are those for 48 hours, as compared to those for 6 hours. *P < 0.01, ^+++^*P *< 0.001.

The proliferative potential of isolated fibroblasts was quantified using the crystal violet assay (Figure 3). In control subjects, central fibroblasts had a significantly lower proliferation potential (1.72 ± 0.46) than distal fibroblasts (2.80 ± 0.72) (p < 0.01). No such difference was observed for fibroblasts from COPD patients. Distal fibroblasts from COPD patients had a significantly lower proliferation potential (2.07 ± 0.27) than distal fibroblasts from control subjects (2.80 ± 0.72) (p < 0.01). No difference was seen between central fibroblasts from COPD patients and control subjects.

### Basal proteoglycan production

**Figure 4:**
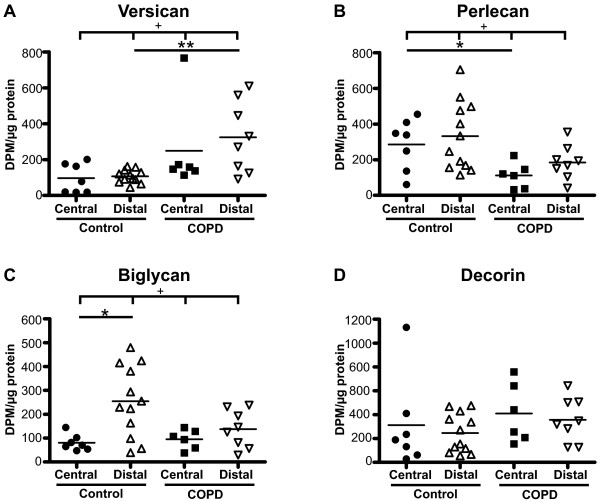
**Basal proteoglycan production**. Fibroblasts were incubated for 24 hours in cell medium containing 0.4% serum, and proteoglycan content in medium was determined by the ability to incorporate [S^35^]-sulphate into glycosoaminoglycan side chains. Values were related to protein concentration in the respective cell layers to account for variability in cell numbers. Individual proteoglycans were separated and quantified on SDS-PAGE gels to determine the contribution of each to the total proteoglycan content. Graph **A **shows production of versican, **B **perlecan, **C **biglycan, and **D **decorin. * *P *< 0.05, ** *P *< 0.01, ^+ ^*P *< 0.05, ^++ ^*P *< 0.01 and ^+++ ^*P *< 0.001.

The basal proteoglycan production was investigated, as shown in Figure 4. In control subjects, distal fibroblasts had a significantly higher production of biglycan than central fibroblasts (255 ± 43 vs. 81 ± 13) (p < 0.05). No such difference was observed for fibroblasts from COPD patients. Distal fibroblasts from COPD patients had a significantly higher production of versican than distal fibroblasts from control subjects (324 ± 198 vs. 90 ± 47) (p < 0.01). Perlecan production was significantly lower in central fibroblasts from COPD patients (111 ± 29) than in fibroblasts from control subjects (285 ± 55) (p < 0.05). There were no significant differences between the groups in the basal production of decorin.

### TGF-β1-induced proteoglycan production

**Figure 5:**
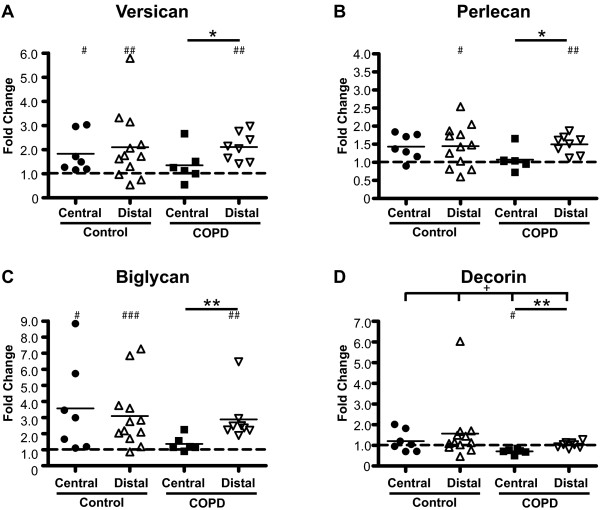
**Proteoglycan production after TGF-β1 stimulation**. Fibroblasts were incubated for 24 hours in cell medium containing 0.4% serum and 10 ng/ml TGF-β_1_, and proteoglycan content in medium was determined by the ability to incorporate [S^35^]-sulphate into glycosoaminoglycan side chains. Values were related to protein concentration in the respective cell layers to account for variability in cell numbers. Individual proteoglycans were separated and quantified on SDS-PAGE gels to determine the contribution of each to total proteoglycan content. Each point represents the fold change after TGF-β_1 _stimulation compared to basal levels. Dashed lines show the basal level of production of each proteoglycan. Graph **A **shows relative change of versican, **B **perlecan, **C **biglycan, and **D **decorin. * *P *< 0.05, ** *P *< 0.01 and ^+ ^*P *< 0.05. # < 0.05, ## < 0.05 and ### < 0.001 indicate significant differences in the proteoglycan production after TGF-β_1 _stimulation compared to basal levels for each group.

Fibroblasts were stimulated with TGF-β_1 _for 24 hours and the proteoglycan production was quantified and compared to basal levels (Figure 5). In central fibroblasts from control subjects TGF-β_1 _induced significant increases in the production of versican (2.1-fold) and biglycan (3.6-fold). In distal fibroblasts from control subjects TGF-β_1 _induced significant increases of versican (1.8-fold), perlecan (1.4-fold) and biglycan (3.1-fold). However, TGF-β_1 _induced a significant decrease of decorin (1.4-fold) but no change in the production of the other proteoglycans in central fibroblasts from COPD patients. In distal fibroblasts from COPD patients, TGF-β_1 _induced significant increases in the production of versican (2.1-fold), perlecan (1.5-fold) and biglycan (2.9-fold). Distal fibroblasts from COPD patients had a higher TGF-β_1_-response in the production of versican (p < 0.05), perlecan (p < 0.05), biglycan (p < 0.01), and decorin (p < 0.01) than central fibroblasts from COPD patients. No such difference was observed between distal and central fibroblasts from control subjects.

### Proteoglycan production profiles

**Figure 6:**
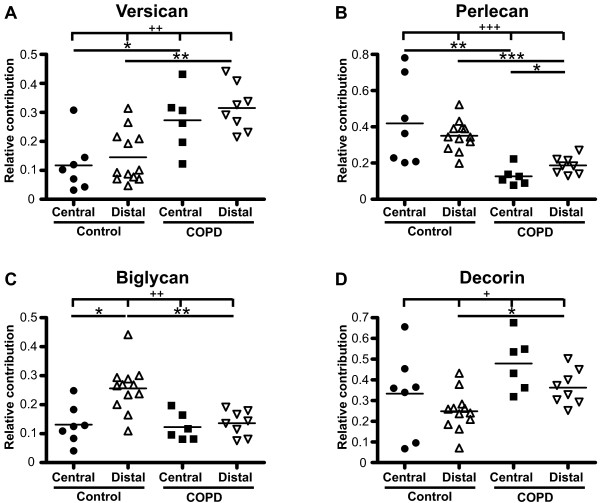
**Profile of proteoglycan production**. Fibroblasts were incubated for 24 hours in cell medium containing 0.4% serum, and individual proteoglycans were separated and quantified on SDS-PAGE gels to determine the contribution of each to the total proteoglycan production. Each point shows the relative contribution of individual proteoglycans to the total proteoglycan content defined as the sum of versican, perlecan, biglycan and decorin (set as 1). Graph **A **shows versican, **B **perlecan, **C **biglycan, and **D **decorin. * *P *< 0.05, ** *P *< 0.01, *** *P *< 0.001, ^+ ^*P *< 0.05, ^++ ^*P *< 0.01, ^+++ ^*P *< 0.001.

We next examined the contribution of each proteoglycan to the total proteoglycan production defined as the sum of versican, perlecan, biglycan and decorin (Figure 6). The contribution of perlecan to the total proteoglycan production was significantly higher in distal fibroblasts (0.19 ± 0.02) compared to central fibroblasts (0.13 ± 0.02) from COPD patients (p < 0.05). There was no such difference in fibroblasts from control subjects. However, in control subjects the contribution of biglycan to the total proteoglycan production was significantly higher in distal fibroblasts (0.26 ± 0.02) compared to central fibroblasts (0.13 ± 0.03) (p < 0.05). There was no such difference in fibroblasts from COPD patients. The contribution of versican to the total proteoglycan production was significantly higher in central fibroblasts from COPD patients (0.27 ± 0.04) than in central fibroblasts from control subjects (0.12 ± 0.03) (p < 0.05). There was a similar difference (p < 0.01) between distal fibroblasts from COPD patients (0.31 ± 0.03) and control subjects (0.15 ± 0.03) in the contribution of versican to the total proteoglycan production. The contribution of perlecan to the total proteoglycan production was significantly lower (p < 0.01) in central fibroblasts from COPD patients (0.13 ± 0.02) than from control subjects (0.42 ± 0.09). A similar difference in the contribution of perlecan was recorded between distal fibroblasts from COPD patients (0.19 ± 0.02) and control subjects (0.35 ± 0.02) (p < 0.001). The contribution of biglycan to the total proteoglycan production was also lower in distal fibroblasts from COPD patients (0.14 ± 0.02) compared to from control subjects (0.26 ± 0.02) (p < 0.01). Finally, the contribution of decorin to the total proteoglycan production was higher in distal fibroblasts from COPD patients (0.36 ± 0.03) than from control subjects (0.25 ± 0.03) (p < 0.05).

## Discussion

Our data indicate that fibroblast in central airways are phenotypically different from those present in the alveolar parenchyma. In COPD patients the phenotypes of these fibroblast populations are altered which may have implications for disease development. Enhanced alveolar versican deposition has been reported in tissue sections from COPD patients, and this may have a negative influence on the elastic recoil. We show that this may be due to dysregulated versican production in fibroblasts. Furthermore, data indicates that there may be structural changes linked to altered molecular composition of basement membranes in cartilaginous airways in severe COPD as the perlecan production was lower and perlecan is important for the basement membrane integrity. Surprisingly, central fibroblasts from COPD patients were poor responders to the pro-fibrotic mediator TGF-β_1_.

Our data support the idea that there are unique and phenotypically different fibroblast populations in central airways and in the lung parenchyma [6,7]. Kotaru *et al*. reported morphological differences between central fibroblasts, which were larger and had more projections, and distal fibroblasts, which were thinner and more spindle-shaped [6]. We observed similar differences in fibroblasts from both COPD patients and control subjects. In addition, distal fibroblast had a higher proliferation potential than central fibroblasts from control subjects. A similar difference has been demonstrated in fibroblasts from asthma patients [6]. Furthermore, in the present study, a lower proliferation potential was observed in distal fibroblasts from COPD patients than from control subjects. This is consistent with previous studies [17-19]. In one study, cigarette smoke was shown to reduce the proliferation of fibroblasts [19]. As the COPD patients in this study have been heavy smokers, with a smoking burden of between 25 and 60 pack years, one could speculate that this may contribute to the lower proliferation potential.

Our data show that the production of biglycan was higher in distal fibroblasts than in central fibroblasts from control subjects. This difference was not observed in COPD patients. Interestingly, biglycan has previously been reported to promote cytoskeletal changes resulting in a more migratory phenotype which may be important in wound healing processes [20]. Furthermore, the proteoglycan production response to TGF-β_1 _did not differ between central and distal fibroblasts from control subjects. However, the production of all the investigated proteoglycans was higher in distal fibroblasts than in central fibroblasts from COPD patients following TGF-β_1_-stimulation. It cannot be explained with a dysregulated response in distal fibroblasts as it was similar in fibroblasts from COPD patients and control subjects. A more plausible explanation is that central fibroblasts from COPD patients are poor TGF-β_1_-responders. This is intriguing as bronchial remodeling in COPD often includes deposition of ECM and TGF-β_1 _is believed to be one of the most prominent inducer of matrix production in fibroblasts. Several studies have reported increased expression of TGF-β_1 _in the airway epithelium and submucosa of patients with COPD and this was proposed to be a product of cigarette smoke as control smokers had similar staining pattern [21-23]. One could speculate that bronchial fibroblasts from COPD patients have been adapted to a local milieu with high concentration of TGF-β_1 _and have as a consequence become less responsive. In the present study TGF-β_1 _induced an increase in proteoglycan production in distal fibroblasts from COPD patients. TGF-β_1_-induced transcription of versican, perlecan, and biglycan requires a functional downstream SMAD signalling pathway [24,25]. This contrasts with a recent study reporting defective repair functions, including migration and contraction, in distal fibroblasts from moderate and severe COPD patients compared to control smokers [26]. The defective repair capability was partially explained by dysregulation of the SMAD signalling pathway. The conflicting results may be explained by differences in control groups as we have non-smokers and Togo *et al*. have smokers with similar smoking history as the COPD patients. Furthermore, it cannot be excluded that the use of biopsy material as the source of control fibroblasts in the present study also may contribute to the difference.

To the best of our knowledge, this is the first study to examine the proteoglycan production in both centrally and distally derived fibroblasts from COPD patients. In our study, an alteration in proteoglycan production was observed in fibroblasts from COPD patients. Versican production was higher in distal fibroblasts from COPD patients than in distal fibroblasts from control subjects. This is consistent with a study that reported increased versican mRNA-expression in distal fibroblasts from COPD patients (GOLD stages II and IV) compared to smoking controls [27]. It is also supported by a recent study showing increased immunostaining of versican in alveolar walls and rims in COPD patients (GOLD stages I and II) relative to control subjects [10]. The amount of versican staining was also reported to be negatively correlated to both elastic fiber volume and to FEV_1_. Previous results from the same group showed that cellular modulations that caused a decrease in versican content or the chondroitin-sulfate side chain content of versican enhanced protoelastin synthesis and elastic fiber assembly in smooth muscle cells [28,29]. These results underscore a role for versican as a negative regulator of elastin.

Structural changes in the small airway wall, including basement membranes, have been suggested to contribute to airflow obstruction in COPD [3]. Less is known about the contribution of structural changes to airflow obstruction in larger airways, although an inverse correlation between airway wall thickness and measures of airflow in cartilaginous airways has been reported [4]. In the present study we observed positive staining for decorin and biglycan in the lamina reticularis, which is normally exclusively composed of collagen I, collagen III, collagen V, laminins, fibronectin, and tenascin. In addition, perlecan production was significantly lower in central fibroblasts from COPD patients than in central fibroblasts from control subjects. Perlecan is a large (> 400 kD) multi-domain heparin sulfate proteoglycan, and a component of the lamina densa in basement membranes of airways and vessels [30]. Knockout studies have suggested that perlecan may be important for basement membrane integrity, and loss of perlecan may render laminins more susceptible to proteolysis [31-33].

An interesting observation in the present study was the shift in the relative production of individual proteoglycans. Distally derived fibroblasts from COPD patients had higher production of versican and decorin and a lower production of perlecan and biglycan than distal fibroblasts from control subjects relative to the total amount of proteoglycan production. Surprisingly, these data are consistent with a rat model investigating bleomycin-induced pulmonary fibrosis, which showed that biglycan production was increased early in the fibrotic process and declined over time, while decorin production was enhanced later in this process [34]. In a subsequent study, it was shown that fibroblasts from patients with pulmonary fibrosis produced relatively more decorin compared to the total proteoglycan content than control subjects [35]. These results indicate that the proteoglycan production in distal fibroblasts from severe COPD is similar to that in fibroblasts in a late fibrotic process. One could speculate that signals in the deteriorating environment of the emphysematous lungs stimulate fibroblasts to produce decorin and versican in an attempt restore the tissue, but as versican has a negative effect on the elastic recoil it leads to disease progression instead of resolution.

The present study was based on fibroblasts isolated from lung explants from COPD patients undergoing lung transplantation and from bronchial and transbronchial biopsies from control subjects. As lung tissue from the two groups is acquired from different sources it may be a potential caveat. Transbronchial biopsies are primarily composed of alveolar tissue but there is a risk of contamination with small airway structures. It was reported that distal lung sampling via transbronchial biopsies contains small airways in one in four to eight biopsies [6]. We have carefully evaluated our material and observed small airways in one in twenty to twenty-five biopsies. However, when present, the small airways component only constitutes a small volume of the entire tissue sample. We are therefore confident that the majority of the fibroblast cultures originate from alveolar tissue. The two groups were not age-matched, and it cannot be excluded that some of the observed differences were a result of this difference. Transbronchial biopsy sampling is an invasive and potentially harmful procedure for subjects; thus, it would not be ethically appropriate to sample older subjects. There have been studies reporting age-dependent changes in the composition of glycosoaminoglycan side chains of decorin and heparan sulphate proteoglycans, such as perlecan, but there has been no evidence of age-related quantitative differences in proteoglycan expression [36,37]. The COPD patients in the present study were all ex-smokers while the controls were non-smokers, and it cannot be ruled out that some of the changes seen could have been due to smoking.

## Conclusions

In summary, the present study supports the idea of the existence of phenotypically distinct fibroblast populations in central airways and in the lung parenchyma. In addition, we show that fibroblasts isolated from COPD patients have alterations in proteoglycan production that may have implications for the pathogenesis. Distally derived fibroblasts from COPD patients had higher versican production, which may have a negative influence on the elastic recoil. Centrally derived fibroblasts from COPD patients had lower production of the basement membrane-stabilizing proteoglycan perlecan, and immunostaining of lung sections showed altered deposition of biglycan and decorin in the basement lamina reticularis. These abnormalities in airway basement membrane composition in COPD are likely to have functional consequences and may thus be regarded as a pathogenic feature in the disease.

## Competing interests

OH has received two research grants from AstraZeneca 2006 and 2008. JE serves as consultant to MedImmune Inc. and received research grants from MedImmune and GlaxoSmithKline. CGL has served on advising boards and given paid lectures organized by various pharmaceutical firms (AstraZeneca, BoeringerIngelheim, GlaxoSmithKline, Novartis, Nycomed and UCB) and has received institutional grants from AstraZeneca and GlaxoSmithKline. LE and MD are employed by AstraZeneca. LB, KN and GWT have no competing interests.

## Authors' contributions

OH and KN performed studies on proliferation and proteoglycan production on primary fibroblasts. OH performed the immunostainings, interpreted the data and drafted the manuscript. MD, LB, LE, JE and CGL contributed to the design of the study and to the writing process. GWT initialized the study and had overall responsibility for the design and writing of the study. All authors read and approved the final manuscript.
